# Clinical and Biological Risk Factors Associated with Increased Mother-to-Child Transmission of HIV in Two South-East HIV-AIDS Regional Centers in Romania

**DOI:** 10.3390/medicina58020275

**Published:** 2022-02-11

**Authors:** Simona Claudia Cambrea, Eugenia Andreea Marcu, Elena Cucli, Diana Badiu, Roxana Penciu, Cristian Lucian Petcu, Elena Dumea, Stela Halichidis, Loredana Pazara, Cristina Maria Mihai, Florentina Dumitrescu

**Affiliations:** 1 Faculty of Medicine, “Ovidius” University from Constanta, 900470 Constanta, Romania; roxanapenciu@yahoo.com (R.P.); elenadumea@yahoo.com (E.D.); shalichidis@yahoo.com (S.H.); loredanapazara@yahoo.com (L.P.); cristina2603@yahoo.com (C.M.M.); 2 Clinical Infectious Diseases Hospital from Constanta, 900709 Constanta, Romania; cucli_elena@yahoo.com; 3 Craiova Clinical Hospital of Infectious Diseases and Pneumophthisiology “Victor Babeș”, 200515 Craiova, Romania; dumitrescu_florentina@yahoo.com; 4 Statistical Department, Faculty of Dental Medicine, “Ovidius” University from Constanta, 900470 Constanta, Romania; crilucpetcu@gmail.com; 5 Faculty of Medicine, University of Medicine and Pharmacy Craiova, 200349 Craiova, Romania

**Keywords:** risk factors, hemoglobin, HIV patients, delivery, mother-to-child transmission

## Abstract

*Background and Objectives:* The occurrence of human immunodeficiency virus (HIV) infection in children in Romania has been reported since 1989. This retrospective study was aimed at assessing clinical and biological risk factors for mother-to-child transmission (MTCT) of HIV in two HIV-acquired immune deficiency syndrome (AIDS) Regional Centers (RCs), Constanta and Craiova in Romania. *Materials and Methods:* During the study period (2008–2019), 408 HIV-positive pregnant women, 244 from Constanta RC and 164 from Craiova RC who attended antenatal visits, were included. All HIV-positive pregnant women were under combined antiretroviral therapy (cART) during pregnancy and childbirth, being followedup with their infants up to 18 months after delivery. We investigated the clinical as well as biological risk factorsassociated with increased MTCT of HIV. *Results:* Comparing different variables of HIV-positive pregnant women from the two HIV-AIDS CRs, we find that there are significant differences between the mean value of hemoglobin, CD4 level, environmental area, marital and amniotic membranes status, and HIV patient stage in the last trimester of pregnancy (*p* < 0.05), but without any differences in mother’s mean age, education level, type of delivery, breastfeeding, the duration of cART administration, HIV viral load, and survival rate. *Conclusions:* In 408 HIV-positive pregnant women followed up at two HIV-AIDS RCs in Romania, the most important clinical and biological risk factors associated with increased MTCT of HIV are represented by anemia, CD4 level, and HIV patient stage.

## 1. Introduction

Since the first reported case of acquired immunodeficiency virus (AIDS) in the United States in 1981, more than 56 million people worldwide have been infected with the human deficiency virus (HIV) virus and some 22 million have died of AIDS. The global infection rate is 15.000 people a day, being responsible for over 90% of the source of infection in young children [1]. The risk of transmission from an infected mother to her infant ranges from about 15% to 45%, with the highest rate reported in sub-Saharan Africa [2]. The first case of AIDS announced in a child in Romania was in 1989, with notification since 1990. From that time, Romania was considered a country with a lower prevalence of HIV-AIDS [3]. Interestingly, mother-to-child-transmission (MTCT) of HIV infection in Constanta was evaluated at 25% for the period 2000–2002 in a pilot study conducted in Constanta County [4] with a continuous decrease until 2004 at 4% [5].

The main risk factors affecting MTCT of HIV can be divided into five categories: maternal factors (e.g., maternal immunologic status, anti-retroviral treatment), virologic factors (e.g., viral load), obstetric factors (e.g., traumatic delivery, chorioamnionitis, caesarean section), fetal factors (e.g., prematurity), and infant factors (e.g., immune status, breastfeeding, nutrition) [6]. Ngwende and contributors [7] show two major factors driving HIV infection in infants: breastfeeding and combined antiretroviral therapy (cART) prophylaxis. This study found out that children born to mothers with a low CD4 count were at risk of HIV infection. A low CD4 count showed an indicator of high viral load. Other studies had similar findings reporting that women with CD4 counts less than 200 cells/μL were five times more likely to transmit HIV during breastfeeding [8,9]. Liu et al. [10] describe that high maternal viral load, measured at delivery, as the strongest risk factor for both in utero and intrapartum transmission of HIV, and CD4+ T cell count, along with clinical stage of infection were also confirmed as significant predictors of transmission. The results of Osorio et al. [11] showed that in rural Mozambique, the main predictor of MCTC was maternal viral load of 1000 copies/mL or more before delivery, as well as fewer than three antenatal care visits. Not being on cART by the first trimester and treatment non-adherence were also related to MTCT of HIV.

Although the newly affective treatment for prevention of MTCT of HIV has been developed, vertical transmission remains the main methodof HIV infection in children. In low-income countries, many improvements in prevention of MTCT of HIV were observed such as the available efficient cART according to HIV-positive pregnant women [12]. In this context, despite efforts to eliminate pediatric HIV infection by 2015, only few countries have successfully eliminated pediatric HIV disease [13].

Considering that the initiation of an early cART for every HIV-infected pregnant woman began to grow, data are still not systematically collected and further reported. However, in recent decades the prophylaxis of MTCT of HIV infection in Romania was a priority and the rate of transmissibility decreased [5].

Finally, most studies support the monitoring of risk factors in MTCT of HIV-infection [14,15,16,17]. Therefore, we developed the present study in order to address the most important clinical and biological risk factors associated with increased MTCT of HIV in two South-East HIV-AIDS Regional Centers (RCs), Constanta and Craiova, in Romania.

## 2. Materials and Methods

### 2.1. Study Design

We conducted a retrospective study on HIV-positive pregnant women in two AIDS RCs that Romania has in terms of treatment and monitoring of HIV-AIDS infection, during the 2008–2019 period, being the most representative HIV-AIDS Centers fromSouth-East of Romania from the total of nine Romanian Centers, that combines a small population-based survey sample with a facility-based sample.

During the study period, in Constanta 269 HIV-positive pregnant women and 215 HIV-positive pregnant women from Craiova attended the day clinic of both hospitals, the Centers being only for HIV-positive pregnant women. From the total of 484, only 408 HIV-positive pregnant women were included in the study, 75 patients having incomplete data and were excluded from the study. From 408 HIV-positive pregnant women, 244 were HIV-positive pregnant women from Contanta RC and 164 HIV-positive pregnant women from Craiova RC. All HIV-positive pregnant women were under cART (p.o. administration) during pregnancy and childbirth, being followedup with their children up to 18 months postpartum.

At enrolment, all HIV-positive pregnant women were interviewed by two HIV trained physicians until the determined sample size of 408 women was reached.

### 2.2. Variables and Measurements

The variables checked in the study were clinical and biological. Clinical variables were collected from the patient’s medical file at the time of delivery: mother’s age, education level, type of delivery, breastfeeding, the duration of cART administration, environmental area (urban or rural area, considering the fact that in rural areas the access to medical facilities is often difficult), marital status, and amniotic membrane. For the survival rate, the women were checked monthly in our day clinic when they came for released treatment. The biological parameters that were followed were: hemoglobin (normal range (n.r.) =12–15 g/dL), using a Konelab Prome 30I device, CD4 T lymphocytes level (at CD4 values < 200 cells/mmc are patients with AIDS-associated immunosuppression; at CD4 = 200–499 cells/mmc are with immunosuppression associated with HIV infection; and at CD4 > 500 cells/mmc are infected with HIV without immunosuppression) using a flow cytometer device, and HIV viral load (undetectable level at <50 copies/mL in the last trimester of pregnancy) using the ribonucleic acid (RNA)-real-time polymerase chain reaction (PCR) technique. Moreover, HIV patient stage (AIDS or HIV in the last trimester of pregnancy) was calculated according to the Centers for Disease Control, Atlanta 1993 [3].

### 2.3. Interventions

All HIV-positive pregnant women were administered cART consisting of triple therapy based on a combination of nucleotide reverse-transcriptase inhibitors (NTRI), associated with non-nucleoside reverse-transcriptase inhibitor (NNRTI) or protease inhibitors (PI) or integrase inhibitors (II).

Patients who underwent acaesarean section received one dose of prophylactic antibiotics (i.e., Cephalosporin) before surgical operation.

Newborns received prophylaxis with ART immediately after delivery for a period of 6 weeks. The ART used in newborns was zidovudine (ZDV) and lamivudine (3TC) with/without Nevirapine, dose adapted with their weight, gestational age, and mother’s HIV viral load in the last trimester of pregnancy, according to theEuropean AIDS Clinical Society guidelines [18].

### 2.4. Data Collection and Analysis

Follow up data for eligible HIV-positive pregnant women were obtained from women themselves. The study was designed to allow for periodic requisitioning of study participants, at birth until 18 months postpartum.

Patients: Blood samples of 408 HIV-positive pregnant women were collected from the two National RCs for HIV-AIDS from Constanta and Craiova RCs, Romania during the 2008–2019 period. Pregnant women were already diagnosed with HIV infection or tested for HIV starting from the 1st trimester of pregnancy. All HIV-positive eligible pregnant women took cART during pregnancy.

Laboratory testing in pregnant women: Confirmation of HIV reactive serum samples was achieved using a flow cytometer device (AQUIOS) for CD4 and Western Blot HIV-1/2 (from National Institute for Medical-Military Research-Development, Cantacuzino, Bucharest from Romania) or RT-PCR (GeneXpert).

Laboratory testing in newborns: HIV testing of infants was performed in the first 24 h after delivery, then at 2, 4, 6, 12, and 18 months using RT-PCR.

The statistical analysis was performed using IBM SPSS statistics software version 23. The procedures used were descriptive statistics, graphs, and parametric/nonparametric statistical tests. Data are presented as mean ± standard deviation (SD) for continuous variables in the case of symmetric distributions, median and interquartile range (IQR) for continuous variables in the case of skewed distributions, or as frequencies and percentages for categorical variables. The normality of the continuous data was estimated with Kolmogorov–Smirnov tests of normality. For hypotheses testing, independent samples *t*-Test, independent samples Mann Whitney U test, independent samples median test, Chi-square test of association, and Chi-squared test for the comparison of two proportions were used depending on the types of analyzed variables. Kaplan–Meier survival analysis with Logrank test for comparison of survival curves was performed using MedCalc statistics software version 14.8.1. The probability of a Type I error (the significance level α) was set at 0.05. If the test statistic for every conducted test was in the critical region, and the *p*-value was less than or equal to the significance level, we decided to reject the null hypothesis in favor of the alternative hypothesis.

Informed consent was obtained from all participants in the study as well as the Agreement of the Ethics Commission (No. 2535/24.02.2021) for the publication of the data.

## 3. Results

From the total of 244 HIV-positive pregnant women from Constanta RC, only 227 HIV-negative infants were born and from 164 HIV-positive pregnant women monitored in Craiova RC, only 155 HIV-negative infants were born.

Analyzing the data from this study regarding the mother’s age, we found that the mean age of HIV-positive pregnant women from Constanta RC did not differ significantly from the mean age of HIV-positive pregnant women from Craiova RC (24.77 ± 4.85 years vs. 24.87 ± 4.39 years, *p* = 0847, Table 1).

It is already known that in the case of HIV-positive pregnant women with anemia or low levels of CD4 lymphocytes in pregnancy, their products of conception may be affected in terms of intrauterine development. Analyzing these two parameters, we found that there are some differences between the two RCs, as seen in Table 1.

Therefore, we noticed that there are also significant differences between the mean value of hemoglobin level in Constanta RC (10.49 ± 1.41 g/dL) and the mean value of hemoglobin level in Craiova RC (11.41 ± 1.33 g/dL) in the last trimester of pregnancy (*p* < 0.001, Table 1).

The mean values of CD4 T lymphocytes were over 400 cells/mmc in both RCs. We found also significant differences between the mean value of CD4 lymphocytes in Constanta RC (479.64 ± 294.86 cells/mmc) and the mean value of CD4 lymphocytes in Craiova RC (411.16 ± 270.05 cells/mmc) in the last trimester of pregnancy (*p* = 0.018, Table 1).

We did not find any association between the level of education and location (Constanta/Craiova RCs) for HIV-positive pregnant women (*p* = 0.06 > α = 0.05, Chi-square test of association). In total, 125/51.2% of 244 patients from Constanta RC and 100/61.0% of 164 patients from Craiova RC completedprimary level education, 28/11.5% of 244 patients from Constanta RC and 10/6.1% of 164 patients from Craiova RC completed higher education, and 27/11.1% of 244 patients from Constanta RC and 10/6.1% of 164 patients from Craiova RC completedno studies at all. There are no differences in proportions between the two locationsfor any of the above education level categories (*p* > 0.05, Table 2).

We found an association between the environmental area (urban/rural) and location (Constanta/Craiova RCs) for HIV-positive pregnant women (*p* < 0.001 < α = 0.05, Chi-square test of association). The proportion of HIV-positive pregnant women from urban areas in Constanta RC (44.8%, 118 of 244) is significantly different than the proportion of HIV-positive pregnant women from urban areas in Craiova RC (16.5%, 27 of 164) and the proportion of HIV-positive pregnant women from rural areas in Constanta RC (51.6% 126 of 244) is significantly different than the proportion of HIV-positive pregnant women from rural areas in Craiova RC (83.5%, 137 of 164) (*p* < 0.001, Table 2).

Regarding the marital status (married/unmarried) of HIV-positive pregnant women, we noticed an association with their location (Constanta/Craiova RCs) (*p* = 0.043 < α = 0.05, Chi-square test of association). The proportion of HIV-positive pregnant married women from Constanta RC, (54.1%, 132 of 244) is significantly different than the proportion of HIV-positive pregnant married women from Craiova RC (43.9%, 72 of 164) and the proportion of HIV-positive pregnant unmarried women from Constanta RC (45.9%, 112 of 244) is significantly different than the proportion of HIV-positive pregnant unmarried women from Craiova RC (56.1%, 92 of 164) (*p* = 0.043, Table 2).

We found an association between HIV stage (AIDS/HIV) and location (Constanta/Craiova RCs) for HIV-positive pregnant women (*p* < 0.001 < α = 0.05, Chi-square test of association). The proportion of HIV-positive pregnant women with AIDS from Constanta RC (63.1%, 154 of 244) is significantly different than the proportion of HIV-positive pregnant women with AIDS from Craiova RC (23.2%, 38 of 164) and the proportion of HIV-positive pregnant women with HIV from Constanta RC (36.9%, 90 of 244) is significantly different than the proportion of HIV-positive pregnant women with HIV from Craiova RC (76.8%, 126 of 164) (*p* < 0.001, Table 3).

We found an association between the women’s membranes in the moment of delivery (broken/intact) and location (Constanta/Craiova RCs) for HIV-positive pregnant women (*p* = 0.037 < α = 0.05, Chi-square test of association). The proportion of HIV-positive pregnant women with broken membranes from Constanta RC (36.1%, 88 of 244) is significantly different than the proportion of HIV-positive pregnant women with broken membranes from Craiova RC (26.2%, 43 of 164) and the proportion of HIV-positive pregnant women with intact membranes from Constanta RC (63.9%, 156 of 244) is significantly different than the proportion of HIV-positive pregnant women with intact membranes from Craiova RC (73.8%, 121 of 164) (*p* < 0.05, Table 3).

We did not find any association between the birth types (caesarean section/vaginal) and location (Constanta/Craiova RCs) for HIV-positive pregnant women (*p* = 0.067 > α = 0.05, Chi-square test of association). In total, 196/80.3% of 244 patients from Constanta RC and 119/72.6% of 164 patients from Craiova RC underwent caesarean section as type of delivery, 48/19.7% of 244 patients from Constanta RC and 45/27.4% of 164 patients from Craiova RC underwent vaginal type of delivery. There are no differences in proportions between the two locations for any of the above birth types (*p* > 0.05, Table 3).

Regarding breastfeeding, we found no association with the location (Constanta/Craiova RCs) of HIV-positive pregnant women (*p* = 0.544 > α = 0.05, Chi-square test of association). There are no differences in proportions between the two locations for any of the breastfeeding types (*p* > 0.05, Table 3).

In pregnancy, another important factor to evaluate, besides the immunology status together with the CD4 T lymphocytes number determination, is also the evaluation of HIV viral load. For the safety and reduction of MTCT of HIV, this determination must be as close as possible to undetectability.

Analyzing the HIV-positive pregnant women from the two RCs according to the viral load recorded in the last trimester of pregnancy, we noticed that the medians of log (HIV viral load) are the same across categories of RCs (1.70 with IQR = 2.19 in Constanta RC, and 1.70 with IQR = 1.81 in Craiova RC: *p* = 0.759, independent samples median test) and the distribution of log (HIV viral load) is also the same across categories of RCs (*p* = 0.056 independent samples Mann Whitney U test).

Regarding the duration of cART (weeks) during pregnancy, we noticed that the cART medians are the same across categories of Regional Centers (37 weeks with IQR = 18 in Constanta RC, and 36.5 weeks with IQR = 35.75 in Craiova RC: *p* = 0.835, independent samples median test) and the distribution of duration cART (weeks) is also the same across categories of RCs (*p* = 0.756 independent samples Mann Whitney U test).

**Figure 1:**
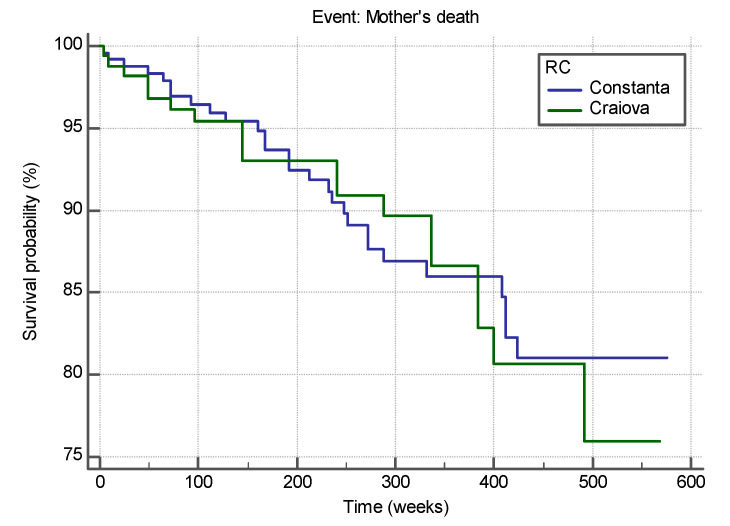
Survival curves of HIV-positive pregnant women from the two RCs, Constanta and Craiova.

The median survival time was 128.06 months (median: 512.25 weeks; 95% CI between 490,848 and 534,211 weeks) for patients in Constanta RC and 125.74 months (502.98 weeks; 95% CI for an average between 490,848 and 534,211 weeks) for patients in Craiova RC, as shown in Figure 1.

The two survival curves do not differ significantly (Chi-squared = 0.024, df = 1, *p* = 0.875 > 0.05, Logrank test for comparison of survival curves), sustaining the similarity in both RCs. The hazard ratio that compares the hazards in the two groups is HR = 0.9545 with a 95% CI for HR = 0.5316 to 1.7139. As value of 1 is included in the 95% CI for HR we state that the estimated relative risk of the event of interest (HIV-positive pregnant women) occurring in Constanta RC does not differ significantly from the estimated relative risk of the event of interest (HIV-positive pregnant women) occurring in Craiova RC.

## 4. Discussion

In the last decade, the AIDS epidemic era has undergone considerable changes and many efforts being implemented, especially in developing countries [19], including rapid tests, or antenatal visits for those patients who were not tested previously, in order to benefit from cART [20].

Before starting to apply cART to HIV-positive pregnant women, many studies showed a higher transmission of the disease from the mother to her infants [21,22,23]. Interestingly, one study revealed that in patients with more advanced disease, ZDV alone had a greatereffect on vertical transmission [24]. All patients treated with ZDV together with their opportunistic HIV infections did not transmit the disease to their infants. All patients received cART (most ZDV and 3TC) together with PIs, being well tolerated during pregnancy [25]. In our study, the compliance with the integrated perinatal management of cART applied shows better results in 94.8% of infants from Constanta RC and 94.5% of infants from Craiova RC.

Important to note, it is relevant nowadays that HIV-positive pregnant woman better understand the obstetrical risks associated with HIV infection, reinforcing that the short duration of broken membranes could be associated with less vertical transmission [26].

Furthermore, there was no other association between mode of delivery and HIV infection in our study, although no HIV transmission was registered in 196 patients from Constanta RC and 119 patients from Craiova RC who underwent elective cesarean section. We believe that elective cesarean section should be reserved to those patients with a higher viral load or those who presented late in delivery without any cART treatment, or with poor adherence at cART [27,28,29,30], considering that caesarean scars could lead to other diseases such asendometriosis [31] in the future, which could causeinfertility, chronic pelvic pain, dysmenorrhea, and dyspareunia [32].

Into our HIV education program, patients have been taught about the syrup and storage administration, together with their infant dose adjustment assessment. In both Constanta and Craiova RCs, the involvement of non-governmental organizations in monitoring and education programs, supports the efforts of physicians in preventing MTCT of HIV infection. However, despite this program implementation, two mothers from Constanta RC and six from Craiova RC breastfed their infants due to socio-cultural factors; such decision tobreastfeed must be taken into consideration as a difficult problem for HIV patients in most countries, where breastfeeding represents a cultural norm [33] such as different lifestyles with foods and more emphasison effective antioxidant and functional ingredients from natural dietary sources [34,35,36].

In a comparative study conducted between 2006 and 2012 on pairs of HIV-positive mothers and their newborns in two RCs in Romania (i.e., Bucharest and Constanta), a series of differences were noticed between the two RCs. In the study performed by Tudor et al. [5], differences were found regarding the transmission rate of HIV infection (16.8% in Bucharest compared to 6.2% in Constanta, *p* = 0.002), type of birth (in Bucharest approximately 1/3 of the mothers gave birth vaginally, *p* < 0.001), but without any differences in terms of mother’s age, which are similar results toour study.

Although clinical trials have been conducted in large cohorts of HIV-positive pregnant women and it has been thought that certain factors such as maternal age or gestational age may influence the number of CD4 lymphocytes in pregnancy, this has not been examined previously. The mean values of CD4 in HIV-positive pregnant women examined in our study were similar to the mean values of pregnant women from other studies [37,38].

Globally, HIV infection accounted for 6–10% of all maternal deaths, most of which occurred in sub-Saharan Africa. Perinatal mortality in HIV-positive pregnant women is 2–10 times higher compared to HIV-negative pregnant women. Some of these deaths can be prevented by treating the associated co-infections and HIV infection with cART [39].

During the 2008–2013 period, one study from Constanta RC showed a mortality among HIV-positive mothers of 7.2%, much smaller compared to 11.4% recorded in our study [37,40]. Moreover, we supposed that the total viral suppression (below 50 copies/mL for this study) shows to have a higher protective effect against MTCT of HIV [41,42].

In our study, a difference was also found between environmental areas of HIV-positive pregnant patients, more women being from rural areas in both Constanta and Craiova RCs (126/51.6% of 224 vs. 137/83.5% of 164), which could be explained by a higher number of infections due to broken membranes at the time of delivery (88/36.1% of 244 for Constanta RC and 43/26.2% of 164 for Craiova RC) along with educational level, the majority of HIV-positive pregnant women havingan elementary school education level (125/51.2% of 244 from Constanta RC vs. 100/61% of 164 from Craiova RC).

It was also showed that HIV-positive patients that are unmarried have a higher risk of MTCT of HIV [43]. Another study undertakenin sub-Saharan Africa found a lower hemoglobin level (i.e., < 11.7 g/dL) during pregnancy to bea risk for MTCT of HIV [44,45], which is in accordance with our study. The mean values of Hb werestatistically different for both RCs (10.49 ± 1.41 for Constanta RC vs. 11.41 ± 1.33 for Craiova RC, *p* < 0.001). Therefore, anemia could represent an important clinical risk factor in HIV-infected patients, especially in pregnant women, who also have low immunity [46].

The strengths of the study are represented by the experience of the two large RCs in South-East Romania, monitoring patients for a period of 10 years, collecting multiple clinical and biological data and tracking the dynamics of some of them. The weaknesses of this study are represented by women who presented incomplete clinical and biological data and those who were lost throughout the surveillance period and were excluded from the study.

Finally, this report could represent an approach for better providing specific bias for independent factors such as infant feeding practices, mode of delivery, the duration of cART administration, and living conditions of HIV-positive pregnant women.

## 5. Conclusions

Comparing different parameters of HIV-positive pregnant women from the two RCs together with their infants, we found that there are significant differences between the mean value of hemoglobin, CD4, environmental area, marital status, amniotic membrane status, and socio-economic status. From 408 mothers followed up at two HIV-AIDS RCs, Constanta and Craiova inRomania, the most important clinical and biological risk factors associated with increased MTCT of HIV are represented by anemia, CD4 level, and HIV patient stage.

## Figures and Tables

**Table 1:** HIV-positive pregnant women’s parameters in the last trimester of pregnancy.

		Regional Centers (*n* = 408)		*p*
		Constanta (*n*_1_ = 244)	Craiova (*n*_2_ = 164)	
		*n*_1_	*n*_2_	
Mother’s age (years)	Mean	24.77	24.87	0.847
	Standard deviation	4.85	4.39	
Hemoglobin level (g/dL)	Mean	10.49	11.41	<0.001
	Standard deviation	1.41	1.33	
CD4 (3rd trimester of pregnancy)	Mean	479.64	411.16	0.018
	Standard deviation	294.86	270.05	

**Table 2:** Distribution ofHIV-positive pregnant women in both Constanta and Craiova RCs according to educational level, environmental area, and marital status.

		Regional Centers (*n* = 408)		*p*
		Constanta (*n*_1_ = 244)	Craiova (*n*_2_ = 164)	
		*n* (% Out of *n*_1_)	*n* (% Out of *n*_2_)	
Educational level	No education	27 (11.1)	10 (6.1)	0.060
	Elementary school	125 (51.2)	100 (61)	
	High school	64 (26.2)	44 (26.8)	
	Higher education	28 (11.5)	10 (6.1)	
Environmental area	Urban	118 (48.4)	27 (16.5)	<0.001
	Rural	126 (51.6)	137 (83.5)	
Marital status	Unmarried	112 (45.9)	92 (56.1)	0.043
	Married	132 (54.1)	72 (43.9)	

**Table 3:** Distribution ofHIV-positive pregnant women in both Constanta and Craiova RCs according to HIV patient scheme (3rd trimester of pregnancy), membranes, delivery, and breastfeeding variables.

		Regional Centers (*n* = 408)		*p*
		Constanta (*n*_1_ = 244)	Craiova (*n*_2_ = 164)	
		*n* (% Out of *n*_1_)	*n* (% Out of *n*_2_)	
HIV patientstage (3rd trim. of pregnancy)	AIDS	154 (63.1)	38 (23.2)	<0.001
	HIV	90 (36.9)	126 (76.8)	
Membranes	Broken	88 (36.1)	43 (26.2)	0.037
	Intact	156 (63.9)	121 (73.8)	
Delivery	Caesarean section	196 (80.3)	119 (72.6)	0.067
	Vaginal	48 (19.7)	45 (27.4)	
Breastfeeding	Yes (breastfeeding)	12 (4.9)	6 (3.7)	0.544
	No (formula)	232 (95.1)	158 (96.3)	

## Data Availability

The data of this report are available from the corresponding authors upon request.

## References

[1] Vieira I.T., Harper P.R., Shahani A.K., de Senna V. (2003). Mother-to-child transmission of HIV: A simulation-based approach for the evaluation of intervention strategies. J. Oper. Res. Soc..

[2] Bulterys M., Lepage P. (1998). Mother-to-child transmission of HIV. Curr. Opin. Pediatr..

[3] CDC (1992). 1993 Revised Classification System for HIV Infection and Expanded Surveillance Case Definition for AIDS among Adolescents and Adults. MMWR.

[4] Cocu M., Thorne C., Matusa R., Tica V., Florea C., Asandi S., Giaquinto C. (2005). Mother-to-child transmission of HIV infection inRomania: Results from an education and prevention programme. AIDS Care.

[5] Tudor A.M., Mărdărescu M., Petre C., Neagu Drăghicenoiu R., Ungurianu R., Tiliscan C., Otelea D., Cambrea S.C., Tănase D.E., Schweitzer A.M. (2015). Birth outcome in HIV vertically-exposed children in two Romanian centers. Germs.

[6] Illif P.J., Piwoz E.G., Tavengwa N.V., Zunguza C.D., Marinda E.T., Nathoo K.J., Moulton L.H., Ward B.J., Humphrey J.H., ZVITAMBO Study Group (2005). Early exclusive breast feeding reduces the risk of postnatal HIV-1transmission and increases HIV free survival. AIDS.

[7] Ngwende S., Gombe N.T., Midzi S., Tshimanga M., Shambira G., Chadambuka A. (2013). Factors associated with HIV infection among children born to mothers on the prevention of mother to child transmission programme at Chitungwiza Hospital, Zimbabwe, 2008. BMC Public Health.

[8] Semba R.D., Kumwenda N., Hoover D.R., Taha T.E., Quinn T.C., Mtimavalye L., Biggar R.J., Broadhead P.G., Miotti L.J., Sokoll L.J. (1999). Human immunodeficiency virus load in breast milk, mastitis andmother to child transmission of HIV type 1. J. Infect. Dis..

[9] Read D.J.S., Breastfeeding and HIV International Transmission Study Group (2004). Late postnatal transmission of HIV-1 in breastfed children: An individual patent data meta-analysis. J. Infect. Dis..

[10] Liu J.F., Liu G., Li Z.G. (2017). Factors responsible for mother to child transmission (MTCT) of HIV-1—A review. Eur. Rev. Med. Pharmacol. Sci..

[11] Osorio D., Munyangaju I., Nacarapa E., Muhiwa A., Nhangave A.V., Ramos J.M. (2021). Mother-to-child transmission of HIV infection and its associated factors in the district of Bilene, Gaza Province—Mozambique. PLoS ONE.

[12] United Nations International Children’s Emergency Fund (2016). For Every Child, End AIDS—Seventh Stocktaking Report.

[13] Joint United Nations Programme on HIV/AIDS (2016). Global AIDS Response Progress Reporting 2016.

[14] Rollins N., Mahy M., Becquet R., Kuhn L., Creek T., Mofenson L. (2012). Estimates of peripartum and postnatal mother-to-child transmission probabilities ofHIV for use in Spectrum and other population-based models. Sex. Transm. Infect..

[15] Remera E., Mugwaneza P., Chammartin F., Mulindabigwi A., Musengimana G., Forrest J.I., Mwanyumba F., Kondwani N., Condo J.U., Riedel D.J. (2021). Towards elimination of mother-to-child transmission of HIV in Rwanda: A nested case-control study of risk factors for transmission. BMC Pregnancy Childbirth.

[16] McConnell M.S., Stringer J.S.A., Kourtis A.P., Weidle P.J., Eshleman S.H. (2007). Use of single-dose nevirapine for the prevention of mother-to-child transmission of HIV-1: Does development of resistance matter?. Am. J. Obstet. Gynecol..

[17] Hoffman R.M., Black V., Technau K., van der Merwe K.J., Currier J., Coovadia A., Chersich M. (2010). Effects of highly active antiretroviral therapy duration and regimen on risk for mother-to-child transmission of HIV in Johannesburg, South Africa. J. Acquir. Immune Defic. Syndr..

[18] European AIDS Clinical Society Guidelines Version 11.0. https://www.eacsociety.org/media/final2021eacsguidelinesv11.0_oct2021.pdf.

[19] Chewe L. (2000). Strategies for prevention of Mother-to-child transmission of HIV. Reprod. Health Matters.

[20] Bulterys M., Jamieson D.J., O’Sullivan M.J., Cohen M.H., Maupin R., Nesheim S., Webber M.P., Van Dyke R., Wiener J., Branson B.M. (2004). Rapid HIV-1 Testing during Labor: A Multicenter Study. JAMA.

[21] Abiodun M.O., Ijaiya M.A., Aboyeji P.A. (2007). Awareness and knowledge of mother-to-child transmission of HIV among pregnant women. J. Natl. Med. Assoc..

[22] Jourdain G., Mary J.Y., Le Coeur S., Ngo-Giang-Huong N., Yuthavisuthi P., Limtrakul A., Traisathit P., Mcintosh K., Lallemant M. (2007). Risk factors for in utero or intrapartum mother-to-child transmission of human immunodeficiency virus type 1 in Thailand. J. Infect. Dis..

[23] Domingues R.M.S.M., Leal M.D.C., Pereira A.P.E., Ayres B., Sánchez A.R., Larouzé B. (2017). Prevalence of syphilis and HIV infection during pregnancy in incarcerated women and the incidence of congenital syphilis in births in prison in Brazil. Cad. Saude Pulica.

[24] El Beitune P., Duarte G., Quintana S.M., Figueiró-Filho E.A., Marcolin A.C., Abduch R. (2004). Antiretroviral therapy during pregnancy and early neonatal life: Consequences for HIV-exposed, uninfected children. Braz. J. Infect. Dis..

[25] Lambert J.S., Moye J. (1999). Current issues in the immunoprophylaxis of vertical transmission of HIV. BioDrugs.

[26] Belachew A., Tewabe T., Malede G.A. (2020). Prevalence of vertical HIV infection and its risk factors among HIV exposed infants in East Africa: A systematic review and meta-analysis. Trop. Med. Health.

[27] Read J.S. (2000). Cesarean section delivery to prevent vertical transmission of human immunodeficiency virus type 1. Associated risks and other considerations. Ann. N. Y. Acad. Sci..

[28] Kennedy C.E., Yeh P.T., Pandey S., Betran A.P., Narasimhan M. (2017). Elective cesarean section for women living with HIV: A systematic review of risks and benefits. AIDS.

[29] ACOG Committee Opinion Labor and Delivery Management of Women with Human Immunodeficiency Virus Infection. https://www.acog.org/-/media/project/acog/acogorg/clinical/files/committee-opinion/articles/2018/09/labor-and-delivery-management-of-women-with-hiv-infection.pdf.

[30] Kourtis A.P., Ellington S., Pazol K., Flowers L., Haddad L., Jamieson D.J. (2014). Complications of cesarean deliveries amongHIV-infected women in the United States. AIDS.

[31] Penciu R.C., Postolache I., Steriu L., Izvoranu S., Tica A.A., Mocanu I.D., Sarbu V., Deacu M., Tica I., Baltatescu G.I. (2020). Is there a relationship in-between ovarian endometriosis and ovarian cancer? Immunohistochemical profile of four cases with coexisting ovarian endometriosis and cancer. Rom. J. Morphol. Embryol..

[32] Penciu R.C., Postolache I., Tica A., Steriu L., Izvoranu S., Mocanu D., Sarbu V., Tica O., Deacu M., Baltatescu G. (2019). Gynecological symptoms correlated with immunohistochemical aspects of endometriosis and adenomyosis. Rev. Chim..

[33] Samburu B.M., Kimiywe J., Young S.L., Wekesah F.M., Wanjohi M.N., Muriuki P., Madise N.J., Griffiths P.L., Kimani-Murage E.W. (2021). Realities and challenges of breastfeeding policy in the context of HIV: A qualitative study on community perspectives on facilitators and barriers related to breastfeeding among HIV positive mothers in Baringo County, Kenya. Int. Breastfeed J..

[34] Zia-Ul-Haq M., Ahmad S., Stankovic M.S., Sultan M.T., Imran I., Velter V., Badiu D., Halichidis S., Hangan T. (2014). Antimicrobial and antioxidant potential of Ipomoea hederacea. Rev. Farm..

[35] Hangan L.T., Carabineanu A., Badiu D., Crainiceanu Z., Cumpanas A., Bardan R., Ciurlea S., Oancea A., Navolan D.B. (2016). The benefits of olive oil compounds in healing burned skin lesions. Rev. Chim..

[36] Maugeri A., Barchitta M. (2020). How Dietary Factors Affect DNA Methylation: Lesson from Epidemiological Studies. Medicina.

[37] Cambrea S.C., Tănase D.E., Ilie M.M., Diaconu S., Marcaş C., Carp D.S., Halichidis S., Petcu L.C. (2013). Can HIV infection during pregnancy cause an intrauterine growth restriction?. BMC Infect. Dis..

[38] Akinbami A.A., Gbadegesin A., Ajibola S.O., Uche E.I., Dosunmu A.O., Sobande A., Uche E. (2015). Factors influencing CD4 cell count in HIV-positive pregnant women in a secondary health center in Lagos, Nigeria. HIV/AIDS-Res. Palliat. Care.

[39] Lathrop E., Jamieson D.J., Danel I. (2014). HIV and maternal mortality. Int. J. Gynecol. Obstet..

[40] Cambrea S.C., Popescu G.G., Resul G., Petcu C.L. (2019). The spectrum of infectious diseases hospital mortality by HIV status. Acta Med. Mediterr..

[41] Kara A., Bayram N., Devrim I. (2015). Postpartum antiretroviral prophylaxis with zidovudine, lamivudine, and nevirapine during intrapartum HIV infection. J. Pediatr. Infect..

[42] Woldesenbet S.A., Kufa T., Barron P., Chirombo B.C., Cheyip M., Ayalew K., Lombard C., Manda S., Diallo K., Pillay Y. (2020). Viral suppression and factors associated with failure to achieve viral suppression among pregnant women in South Africa. AIDS.

[43] Mugwaneza P., Lyambabaje A., Umubyeyi A., Humuza J., Tsague L., Mwanyumba F., Mutabazi V., Nsanzimana S., Ribakare M., Irakoze A. (2018). Impact of maternal ART on mother-to-child transmission (MTCT) of HIV at six weeks postpartum in Rwanda. BMC Public Health.

[44] Duri K., Gumbo F.Z., Kristiansen K.I., Kurewa N.E., Mapingure M.P., Rusakaniko S., Chirenje M.Z., Muller F., Stray-Pedersen B. (2010). Antenatal HIV-1 RNA load and timing of mother to child transmission; a nested case-control study in a resource poor setting. Virol. J..

[45] Bobat R., Coovadia H., Coutsoudis A., Moodley D. (1996). Determinants of mother to- child transmission of human immunodeficiency virus type 1 infection in a cohort from Durban, South Africa. Pediatr. Infect. Dis. J..

[46] Harding B.N., Whitney B.M., Nance R.M., Ruderman S.A., Crane H.M., Burkholder G., Moore R.D., Mathews W.C., Eron J.J., Hunt P.W. (2020). Anemia risk factors among people living with HIV across the United States in the current treatment era: A clinical cohort study. BMC Infect. Dis..

